# Syphilitic and Other Cutaneous Diseases Cured without Mercury

**Published:** 1866-05

**Authors:** John M. Cornell


[For the Buffalo Medical and Surgical Journal.]

### Syphilitic and other Cutaneous Diseases cured without Mercury.

BY JOHN M. CORNELL, M. D.

To the Editor of the Buffalo Medical and Surgical Journal:

I thank you for your very excellent Journal which has come for the last three or four years. You will allow me to say: I have been so highly gratified with your article, "Necrosis of the Skull," in your March number, that I venture to send you the following, which you will publish, if you think proper:

You say, "mercury, properly administered, will, in most constitutions, control the disease, (syphilis). It is also sometimes useful in other stages of the disease ; but, it is not a specific, and will not wholly eradicate it from the system at any time. It is capable of good—it is also potent for evil; and as it generally has been, and still is prescribed, quite as often works mischief as benefit."

This, so completely corresponds with my own views and experience, that I venture to give you my treatment of some cutaneous diseases, especially of a syphilitic character. Our profession, I am convinced, has placed too much reliance in mercurial preparations in the syphilitic taint, and they have prescribed it too often, and too carelessly. I have had many cases where I had reason to believe the disease was mercurial, though it had been pronounced syphilitic.

I purpose to show that, in the whole class of cutaneous, and other diseases arising from *syphilitic taint*, other remedies are more certain in the removal of the difficulty, and much more safe for the patient, than mercury.

Mercury, in some of its forms, has been considered *the specific* for this disease in all its stages; though it has generally been admitted, that it was eradicated before this mineral was employed as a medicine, and has often been since, without its use. Happily, both for the credit of the doctor, and the welfare of the patient, the old method of *salivation* is no longer resorted to, even by those who still believe that mercury is necessary in the treatment and cure of the disease in some of its forms.

Having had some experience, during the last twenty years, in treating *diseases of the skin*, whether they had originated from this peculiar malady, or had sprung from other causes, I wish to state my opinion, and the arguments upon which it is founded.

I have used mercurial preparations, and seen them used, in all their forms, in the various stages of the disease above named, and I have yet to find *a solitary case, of a chronic form*, which has been removed, or alleviated even, by the drug now in question, save only in one form hereafter to be named. I look upon the *stillingia sylvatica* as the best vegetable alterative, in this whole class of diseases, in their *chronic* form; and I have great confidence that the physician who perseveres in its use will find his patients improve, and that much more generally, than under the use of arsenic or mercury.

Another medicine in these cases which has proved highly satisfactory in my hands, is the *nitric acid*, given in doses of ten drops three times a day. Thirty years ago this medicine was given much more frequently, in debilitated constitutions, than is at present. In the debility attendant upon these cases, the following is often a serviceable remedy. ℞. Com. tinct. bark, ℥ ij.; sulph. quinia grs. xij.; muriatic acid, gtt. xx. M. Dose, a teaspoonful *ter die.* The old oxygenated muriatic acid was much employed in these diseases many years ago. It probably forms the basis of a preparation, now sold under the name of "*Oxygenated Bitters*," and is a valuable medicine when it *hits the case*, which it probably does not one time in fifty, when purchased and taken at random. I have found the following useful, in some of these old broken-down constitutions. ℞. Ioduret of iron, grs. xxx; castile soap, grs. xxx; alkaline ext. gentian, ʒi. M. Ft. pil. No. xxx. Dose: one pill night and morning.

The *diet drink*, of the pharmacopia, is one of the best medicines for purifying the blood. For the same purpose, the following receipt furnishes an excellent alterative. ℞. Iodide of potassium, ʒi.; iodine, gr. ij.; mucilage of acacia, ℥ iij.; hydrocyanic acid, gtt. xij.; aqua pura, ℥v.; sach., ℥ss. M. Dose: a table-spoonful twice a day, in a wine-glassful of water. The *bromide* may be substituted for the iodide of potassium, as it is equally efficacious, though it requires a longer time to produce its beneficial effects. The only advantage possessed by the latter is, it is cheaper.

If mercury is ever to be employed as an alterative, in these forms of constitutional taint manifested by cutaneous eruptions, the most efficacious form in which I have used it is that of Dr. Channing, named in the U. S. Dispensatory, page 1340, of the edition of 1851 under the name of *iodo-hydrargyrate of potassium.* "The average dose of this remedy is stated by Dr. C. to be one twelfth of a grain three times a day; but, in peculiar constitutions, not more than the forty-eighth, ninety-sixth, or the two hundredth of a grain, daily, can be borne." The testimony of many physicians is much in favor of this medicine as an alterative.

I am by no means alone as it respects treating this whole class of diseases without mercury. In the *New York Journal of Medicine and Collateral Sciences,* I find the following remarks, which I consider very judicious and sensible. They are from the pen of Dr. Scott, and relate to the non-mercurial treatment of syphilis. "Thirty years since," he says, "there was no doctrine in the profession, which was considered to be so well founded as the treatment of syphilis by mercury. In England, none presumed to differ from the opinion of John Hunter, that the disease was incurable without mercury; and not only that the medicine was required to remove the disease itself, but that to cure the disposition to it, and to secure the constitution from its ravages, an extended course of mercury was required. Sir Benjamin Brodie still retains this opinion, and he (Dr. Scott) would not have called the attention of the Society to this subject, had he not observed, in the lately published essays of Sir Benjamin, some remarks, which from so high an authority appeared to him calculated to lead to an injurious line of practice. Every now and then a dissenting voice had been raised against the mercurial doctrine, but the profession in general, adhere to the opinion of John Hunter."

Dr. Scott's own experience is related as follows:—"In 1813, he was placed, for a short time, in Columbo, in charge of the venereal wards, in which the cases were all treated with mercury. Many of them, he found, were well in a few days; others in five or six, others in three weeks; periods too short to warrant the conclusion that they were venereal. They were, therefore, set down as cases pseudo-syphilis. The number of these cases increased with the field of experience; and, in a few years, the use of mercury was gradually resigned in almost every case of local disease. The *secondary* symptons were few and slight, and never required an extended course of mercury. The same plan of treatment was adopted by them, and in a few years, Dr. Scott, then garrison surgeon at Point de Galle, entirely abandoned the use of mercury. In 1818 and 1819, Dr. Scott became acquainted with the results of the investigations which had been carried on in England, and since that time, had abandoned the use of mercury, as a specific. He had found many cases in which it was required, as an alterative. Dr. S. stated that he considered every case of local disease curable without mercury; and that, under such treatment, the secondary symptoms, when they did occur, were slight, and easily managed. Dr. S., in the course of his remarks, described the miserable victims who were constantly found in military hospitals, at the time mercury was used, affected by extensive ulcerations, nodes, &c., who furnished a considerable number of the invalids, and many deaths. Since mercury was abandoned, such cases had disappeared from the hospitals."

Dr. Maclagan expressed his satisfaction that Dr. Scott coincided in the views he (Dr. M.) had long entertained on this subject. His confidence in mercury, as a specific in syphilis, had been first shaken when, after he was a graduate in medicine, he attended, for some months, the Lock Hospital, in London, under Mr. John Pearson. There, every variety of form in the disease presented itself; but, in very many cases, seemed to be aggravated, rather than benefited, by the mercurial course. Dr. Pearson often expressed doubts, whether, in many constitutions, the use of mercnry had not been more injurious than beneficial. Dr. Maclagan had seen Portuguese soldiers cured of the primary form of the disease by topical remedies alone, or merely by the addition of Lisbon diet and drinks, and sometimes without either. He saw none of those cases of secondary symptoms in an aggravated form, to which his late lamented friend, Dr. Ferguson, has alluded in his paper to the Transactions of the Medical und Chirurgical Society of London. Since 1818, Dr. Maclagan, with a few exceptions, where the patient's scruples afforded a full explanation, demanding its modified use, had adhered to the non-mercurial plan of treatment, both in dispensary and private practice; and, in no instance, has he had reason to regret it. Many, who were then so treated, are his patients still; fathers of families, enjoying, as well as their offspring, excellent health, and without the occurrence, in the period of thirty years, of any secondary symptoms of an aggravated form. On the other hand, he has seen too many cases where the use of mercury, to its full extent, has been productive of constitutional injury of the most serious character.

Dr. Bennett said, "That reports had been made to the Governments of France, Germany and Sweden, of 80,000 cases, treated upon the non-mercurial plan, and their general results were quite in accordance with the experience of Dr. Scott."

I have related the experience of these men upon a point on which I have not myself had an extensive practice, namely, the

*primary* stages of this disease. My experience has been chiefly in those cases of a chronic form, manifesting the disease in what are called secondary or tertiary symptoms, always arising from a *constitutional taint.* Dealing with chronic diseases, of various forms, especiallv with those of the *skin,* I have seen almost all kinds of such cases; and I have known the most aggravated forms of chronic eruptions, upon the head, face, and other portions of the body, wholly removed, and permanently to disappear, under a treatment without a grain of mercury. In some of these cases mercury had been employed, even to salivation, without any obvious benefit. For more than twenty years I have closely watched these peculiarities of skin diseases, and am satisfied that there is a better, safer, and more eligible method of treating them, than by employing either *mercury* or *arsenic.* If this be so, (and I think it can be proved to be) I ask, are we justified in using heroic remedies, which may produce serious injury to our patients, without removing the original disease? Would not their disuse redound to our credit—would it not be another triumph added to the success of our profession, and does not humanity demand a discontinuance of medicines which are really unnecessary, and often productive of the gravest injury to those who entrust their health and life to our hands?

I am happy to corroborate these views by the following quotation from the *London Lancet* of June 27th, 1857: "In a recent visit to the Royal Free Hospital, where a number and variety of syphilitic cases are to be met with, especially of the secondary eruptions, we find they are treated by the administration of stomachic and tonic remedies and good diet, conjoined with the following formula, viz: sulphur, ʒ i; sulphuret of antimony and nitrate of potass, āā gr. v, mixed into a powder, half of which is given night and morning, and persevered in till the eruption disappears, the health is improved, and a cure established. Dr. Marsden has employed this mode of treatment for twenty-seven years, in thousands of cases, and he observes that not one in a hundred instances has he known to return with constitutional symptoms."

I think our profession mistake often in holding on to a routine practice after it has been shown to be erroneous. We are accused of this by irregular practitioners, and, perhaps not, without some just grounds. The writer has lived longer than many who are now active in the profession, and he can well remember, when, to have attempted to treat syphilis without mercury, and pneumonia without venesection, would have been considered, by the faculty, as empirical. Indeed, forty years ago, no physician could have discarded mercury and blood-letting in these diseases, and still have retained his standing with the profession.

I recollect a discussion in Boston, at a meeting of the Suffolk District Medical Society, when one of the oldest members, at a lull, when no one seemed ready to speak, said: "Gentlemen, since I commenced practice, fifty years ago, the *character of diseases* has changed very much. Patients will not bear such treatment *now* as they would *then.*" Some genius, (it might have been an evil one), prompted me to ask, "Doctor, do you really think the *change* has been in the *character* of the diseases, and the *nature* of the patients, or in the *practice?*" Oh! said he, "in *character* of the diseases and the *constitution* of the patients." Probably he thought so.

Now, the sooner the profession can rid itself of such notions, the better will it stand in the world. Indeed, such notions have led some to the falacy that medicine is of no use—that "if it were all sunk in the sea it would be all the better for men, and all the more for the fishes;" than which a greater blunder was never made. How prone we are to go to extremes!

---

Fort Erie, C. W., April 24, 1866.

To the Editor of the Buffalo Medical and Surgical Journal:

Sir—Will you allow me to tender my grateful thanks for the kind and prompt manner in which you and your medical brethren in Buffalo assisted me by your presence and evidence in the late trial for alleged malpractice, brought against me, and permit me also to thank you, particularly for your just and kind notice in the Medical and Surgical Journal, of this most malicious prosecution instituted (as any impartial, common sense person must perceive who reads the evidence, or your editorial remarks on the trial), solely with the view of extortion, and to avoid the payment of a medical bill. The kind and impartial treatment I have received at the hands of the profession, is the more gratifying, from the fact of my being comparatively a stranger and foreigner, and consequently the action of the Faculty must have been educed, not by personal feeling, but by a pure love of justice, and a noble zeal for the honor of our profession. It has entailed upon me a lasting debt of gratitude to my professional brethren in Buffalo, and it will ever be a source of pride and gratification for me to recall the unanimous verdict in my favor of all the surgeons in Buffalo whose names stand high as men of eminence, talent and respectability.

Some explanation may be deemed necessary on account of my not having "carried up" the case and moved for a new trial, but although I had determined to do so, I find the majority of my professional friends feeling satisfied that the caprice of another jury might decide as on the first trial, and result not in any professional injury, but in a loss of time, attention to patients, and to family affairs, which would involve a far greater loss than any pecuniary one they would inflict.

Trusting you will give these few words space in the next issue of your valuable journal,

I have the honor to subscribe myself,

Your obliged friend,

P. Tertius Kempson, M. D.

