# Correction

**Published:** 1866-06

**Authors:** 


CORRECTION.—The article in the *May* number on "Syphilitic and other Cutaneous Diseases cured without Mercury," was written by *William* M. Cornell, M. D., LL. D., of Philadelphia, not by *John* M., as printed by mistake.

